# Clinical characteristics of pyogenic liver abscess patients with multiple organ dysfunction syndrome and development and validation of a predictive model

**DOI:** 10.3389/fcimb.2026.1828061

**Published:** 2026-08-20

**Authors:** Jiaqi Chen, Liyong Zhang, Jinhua Cui, Lingkun Zhang, Jingyi Luo, Kunyu Huang, Zejin Zhao, Ziyu Bai, Zhuqing Zhang, Aijun Yu, Jian Li, Kai Chen

**Affiliations:** 1 Department of Hepatobiliary Surgery, The Affiliated Hospital of Chengde Medical University, Chengde, Hebei, China; 2 Hebei Key Laboratory of Panvascular Diseases, Chengde, Hebei, China; 3 Department of General Surgery (Unit 1), Sinopharm Northern Hospital (The Third Affiliated Hospital of Baotou Medical College), Baotou, Inner Mongolia, China; 4 Department of Clinical Laboratory Services, The Affiliated Hospital of Chengde Medical University, Chengde, Hebei, China

**Keywords:** *Klebsiella pneumoniae*, multiple organ dysfunction, prediction model, pyogenic liver abscess, retrospective study

## Abstract

**Background:**

Pyogenic liver abscess (PLA) is one of the most common and severe infectious diseases encountered in clinical practice. It is characterized by rapid progression, and failure to recognize and treat the condition promptly may readily result in septic shock and multiple organ dysfunction syndrome (MODS), thereby posing a serious threat to the patient’s life. However, clinical research focusing on the development of MODS in patients with liver abscess remains limited. Therefore, this study aimed to develop and validate a predictive model for MODS in patients with PLA, identify associated risk factors and clinical characteristics, and facilitate the identification of high-risk patients at admission and during the early stage of hospitalization, thereby supporting timely clinical decision-making and management.

**Methods:**

A total of 540 patients diagnosed with PLA were retrospectively enrolled for model development. According to the Sepsis-3 criteria, patients were categorized into a MODS group (n = 67) and a non-MODS group (n = 473). Demographic, clinical, laboratory, and imaging data were collected and analyzed. A dual-path feature selection strategy integrating conventional statistics and machine learning was applied, and key variables were identified using the least absolute shrinkage and selection operator (LASSO), the Boruta algorithm, recursive feature elimination (RFE), and the extreme gradient boosting (XGBoost) combined with SHAP (SHapley Additive exPlanations). Independent risk factors were subsequently confirmed using multivariable logistic regression. The resulting predictors showed high consistency across methods and were subsequently used to develop a logistic regression–based prediction model and construct a nomogram. Finally, model performance was evaluated using receiver operating characteristic (ROC) analysis with the area under the curve (AUC), calibration curves, decision curve analysis (DCA), the Hosmer–Lemeshow goodness-of-fit test, and clinical impact curves. To further assess its predictive value, the model was compared with the established quick Sequential Organ Failure Assessment (qSOFA) score. Differences in AUCs were examined using the DeLong test, and DCA was performed to compare clinical net benefit across a range of threshold probabilities. In addition, the net reclassification improvement (NRI) and integrated discrimination improvement (IDI) were calculated to quantify reclassification performance and the incremental predictive value relative to qSOFA.

**Findings:**

Among the 540 patients with PLA included in this study, 67 developed MODS during hospitalization. Multivariable logistic regression identified invasive *Klebsiella pneumoniae* liver abscess syndrome (IKPLAS) (OR = 7.667, 95% CI: 2.397–24.526), septic shock (OR = 4.662, 95% CI: 1.935–11.235), elevated international normalized ratio (INR) (OR = 6.983, 95% CI: 1.274–38.287), and ascites (OR = 13.834, 95% CI: 5.280–36.245) as independent factors associated with MODS. Platelet count was inversely associated with MODS risk (OR = 0.997, 95% CI: 0.994–1.000), suggesting that thrombocytopenia may increase susceptibility to MODS. The predictive model based on these variables demonstrated good discriminative ability, with area under the curve values of 0.826 (95% CI: 0.749–0.903) in the training cohort and 0.749 (95% CI: 0.587–0.911) in the validation cohort. Calibration curves and DCA further supported the model’s accuracy and potential clinical utility. In addition, the model outperformed qSOFA in predicting MODS in patients with PLA.

## Interpretation

The proposed prediction model demonstrated promising performance in identifying patients with PLA at high risk of developing MODS at admission and in the early stages of hospitalization, and may assist clinicians in initiating timely preventive interventions and individualized management strategies.

## 1 Introduction

PLA is a severe infectious disease caused by bacterial invasion of the liver parenchyma, and its global incidence has increased steadily over recent decades. The disease is particularly prevalent in Asian countries, where the reported incidence may reach up to 86 cases per 100,000 population (Kaplan et al., 2004; Chen et al., 2009; Chen et al., 2014; Shi et al., 2018; Rossi et al., 2022; Oliosi et al., 2023). Although advances in antimicrobial therapy, imaging techniques, minimally invasive drainage procedures, and comprehensive supportive care have substantially improved patient survival, PLA remains associated with serious complications, including sepsis, multiple organ dysfunction syndrome, and death, with reported in-hospital mortality rates ranging from 11.6% to 28% (Chen et al., 2008; Le Goff et al., 2025). Among these, MODS is considered a key terminal pathway leading to infection-related mortality, and its pathogenesis involves the interplay of multiple factors, including an amplified inflammatory cascade, impaired tissue perfusion, and dysregulated immune responses (Gourd and Nikitas, 2020; Kozlov and Grillari, 2022). However, PLA is often characterized by an insidious onset and rapid clinical deterioration. If high-risk patients are not promptly identified and appropriately managed, systemic inflammatory responses, intrahepatic and extrahepatic spread of infection, and microcirculatory dysfunction can precipitate MODS within a short period of time (Singer et al., 2016). Previous studies have shown that although the incidence of MODS among patients with PLA is relatively low, once MODS develops, the risk of death increases markedly (Yin et al., 2021). A recent study from France also reported that organ dysfunction, as assessed by the Sequential Organ Failure Assessment (SOFA) score, is a major risk factor for mortality in patients with PLA, further underscoring the close association between organ dysfunction and poor outcomes (Le Goff et al., 2025).

Despite the clinical importance of early risk stratification, disease-specific tools for predicting MODS in patients with PLA remain limited. Consequently, decisions regarding escalation of antimicrobial therapy, intensive monitoring, and early organ-support interventions frequently rely on clinician experience rather than evidence-based risk assessment. Although commonly used scoring systems, such as the National Early Warning Score 2 (NEWS2) (Corfield et al., 2014; Chen et al., 2025) and the Acute Physiology and Chronic Health Evaluation II (APACHE II) (Yelamanchi et al., 2020; Lee et al., 2021), are useful for evaluating overall disease severity and clinical deterioration, they were not specifically developed for PLA-related complications and may therefore provide suboptimal predictive performance in this population.

Moreover, PLA exhibits marked heterogeneity in clinical presentation, inflammatory response, source control status, and microbiological characteristics, particularly in cases involving hypervirulent *Klebsiella pneumoniae* infection (Siu et al., 2012; Shon et al., 2013). These factors further complicate the identification of patients at increased risk for MODS at admission and during the early stage of hospitalization. Accordingly, the present study aimed to develop and validate a clinically applicable prediction model for MODS in patients with PLA. By identifying key predictors of disease progression and integrating them into a clinical prediction model, we sought to improve the early recognition of PLA-associated MODS and, ultimately, patient outcomes.

## 2 Materials and methods

### 2.1 Study design and population

**Figure 1:**
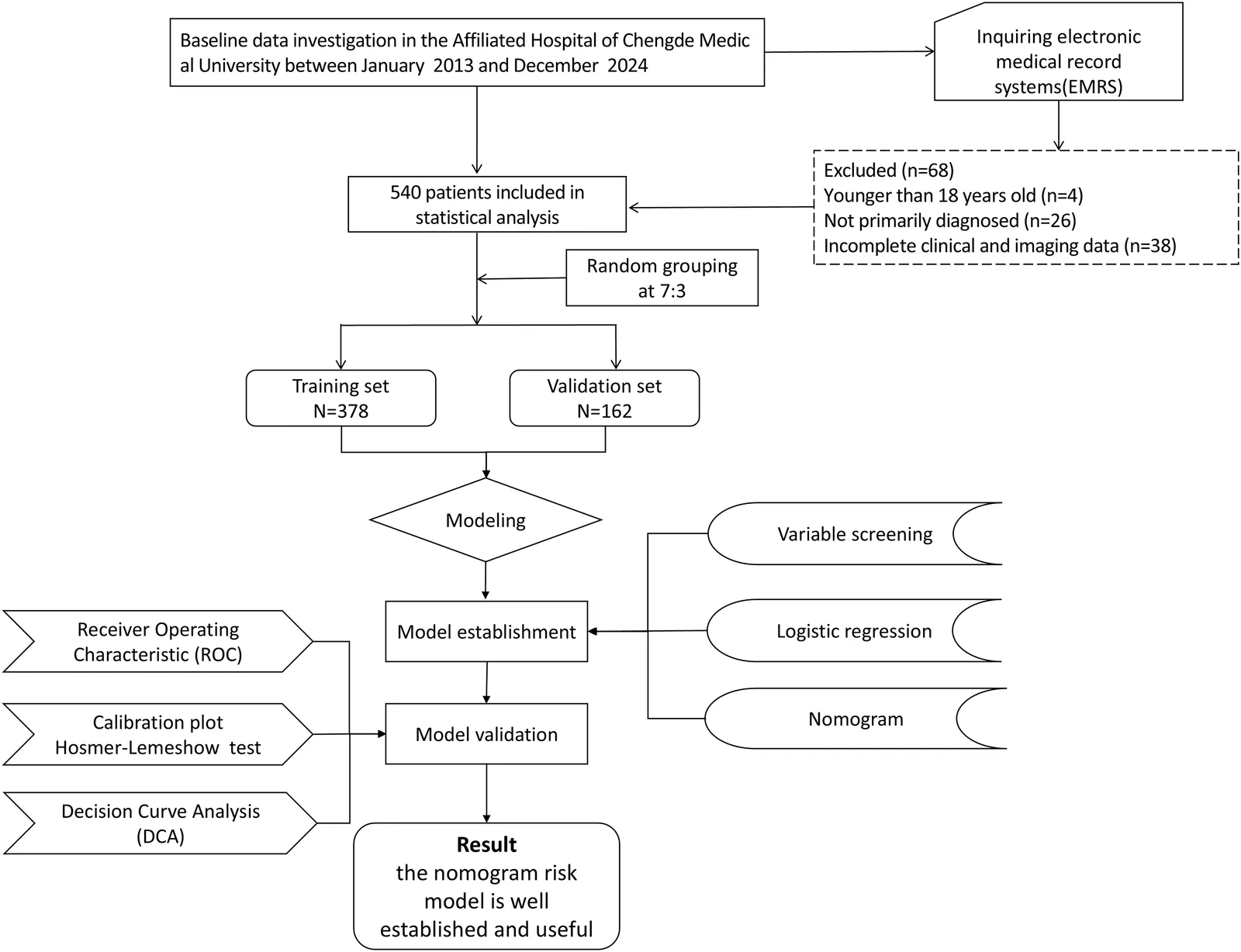
Flow diagram of study design.

This single-center retrospective study was conducted using anonymized clinical data and was approved by the Ethics Committee of the Affiliated Hospital of Chengde Medical University (Approval No. CYFYLL2025552). Owing to the retrospective nature of the study, the requirement for informed consent was waived. We retrospectively screened 608 patients diagnosed and treated for PLA at our hospital between January 2013 and December 2024. The overall study workflow is shown in **Figure 1**.

The inclusion criteria were as follows: (1) first presentation to our hospital after disease onset and age ≥18 years; (2) imaging findings consistent with liver abscess in accordance with the diagnostic criteria for PLA; (3) positive blood and/or abscess fluid culture, or negative culture results but a diagnosis of PLA based on clinical presentation, imaging features, and response to antimicrobial therapy; and (4) a first episode of PLA or recurrence occurring ≥6 months after a previous episode to avoid duplicate enrollment. The exclusion criteria were as follows: (1) hospital stay <24 hours; (2) non-PLAs, including amoebic, fungal, or parasitic abscesses; (3) pre-existing organ dysfunction such as decompensated cirrhosis, chronic renal failure, end-stage heart failure, or chronic respiratory failure at admission; (4) concomitant malignancy, tuberculosis, HIV infection, or long-term immunosuppressive therapy (including corticosteroids or immunosuppressants); and (5) incomplete medical records.

### 2.2 Data collection

Clinical data were independently extracted from the hospital electronic medical record (EMR) system by two investigators and subsequently cross-verified to ensure data accuracy and consistency. Collected variables included demographic characteristics, admission vital signs, comorbidities, and relevant medical histories, including viral hepatitis, cirrhosis, biliary disease, fatty liver disease, hypertension, type 2 diabetes mellitus (T2DM), and hyperuricemia. Infection-related complications and severity indicators, including IKPLAS, pulmonary infection, septic shock, and ascites, were also recorded. Septic shock was defined as shock present at admission or identified within 24 h after admission. IKPLAS was determined according to microbiological, imaging, and clinical evidence obtained during the early hospitalization period. Laboratory tests were obtained within 24 h of admission and included WBC, PLT, Hb, CRP, ALP, INR, and fibrinogen. Microbiological findings were derived from blood and/or abscess aspirate cultures, and the predominant pathogens were recorded. Imaging features were extracted from post-admission computed tomography (CT) reports and included abscess location, number of abscesses, maximal abscess diameter, presence of gas formation, and evidence of perihepatic involvement.

Variables with a missing rate greater than 20%, such as blood urea nitrogen (BUN) and procalcitonin (PCT), were excluded to avoid potential bias caused by excessive imputation. Although these variables are closely associated with renal function and inflammatory response, they were not included in the model because of substantial missingness. For variables with lower missing rates, defined as ≤20%, multiple imputation was performed using the miceforest package in R. The missing data pattern of the relevant variables is shown in **Supplementary Figure 1**.

### 2.3 Outcome measures

The primary endpoint was the development of MODS. According to the Sepsis-3 consensus definition (Singer et al., 2016) and SOFA criteria (Vincent et al., 1996), organ dysfunction was defined as an increase of ≥2 points in the total SOFA score from baseline. In cases in which pre-infection baseline SOFA scores were unavailable, a baseline score of 0 was assumed. For outcome assessment, the highest total SOFA score recorded within the first 24 h after admission was used to represent the most severe early physiologic status. Because SOFA sub-scores for individual organ systems were incompletely documented in the EMR system, organ involvement was not determined exclusively based on sub-scores. Accordingly, the diagnosis of MODS was adjudicated by integrating clinical evidence of dysfunction involving multiple organ systems with the use of organ-support therapies, including mechanical ventilation or other respiratory support, vasopressor administration, and renal replacement therapy, in patients who met the organ-dysfunction criterion (SOFA increase ≥2) within 24 h of admission, and was defined as involvement of ≥2 organ systems. Patients were therefore classified into MODS and non-MODS groups.

The MODS status of each patient was independently assessed by two trained researchers according to predefined criteria. Inter-rater agreement was evaluated using Cohen’s Kappa coefficient. The overall agreement rate was 95.93%, and the Kappa value was 0.815, indicating substantial agreement between the two assessors. For cases with discrepant judgments or ambiguous records, the two researchers jointly reviewed the original medical records and reached a final consensus after discussion with a senior clinician.

All patients received standard antimicrobial therapy and other necessary supportive treatments. Selected patients underwent percutaneous abscess drainage or surgery according to their clinical condition. To evaluate the potential impact of early therapeutic interventions on clinical outcomes, information on whether percutaneous abscess drainage was performed within 24 h after admission and whether empirical broad-spectrum antimicrobial therapy was administered was extracted and compared between the MODS and non-MODS groups (**Supplementary Table 1**).

### 2.4 Feature selection

**Figure 2:**
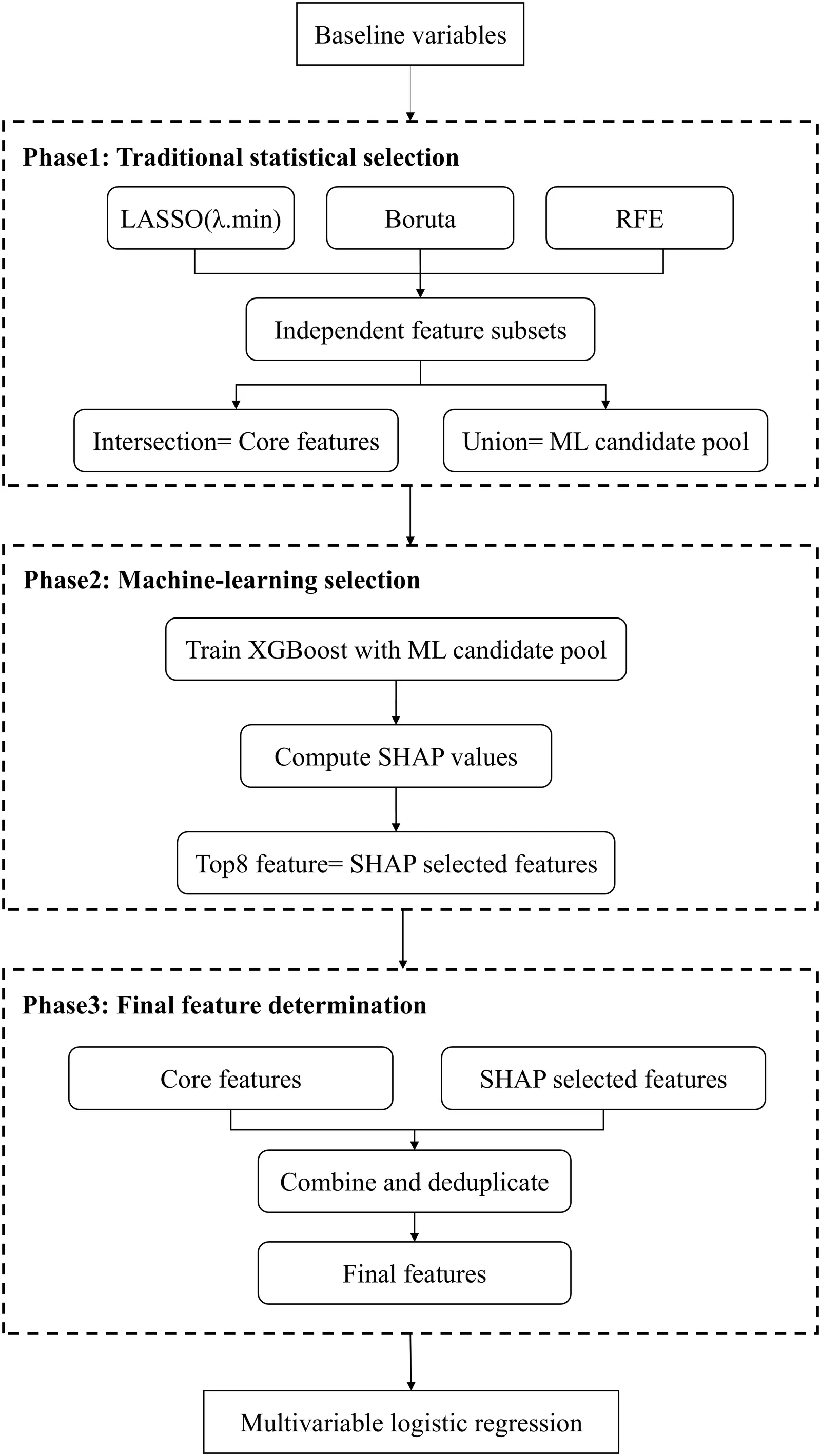
Flow diagram of variable screening.

To improve the robustness, stability, and interpretability of predictor selection, we adopted a dual-track, three-stage feature-selection strategy that integrated conventional statistical approaches with machine learning techniques (**Figure 2**). In the first stage, three complementary feature-selection methods were applied to the training dataset: LASSO regression, Boruta analysis, and RFE. Variables with non-zero coefficients at λ.min in the LASSO model were retained, and each method generated an independent candidate feature set. The intersection of the feature sets identified by all three methods was defined as the core feature set, whereas the union of all selected variables constituted the machine-learning candidate pool. In the second stage, an XGBoost model was developed using the union feature set, and SHAP values were calculated to quantify the marginal contribution of each variable to model performance. Features were ranked according to their mean absolute SHAP values, and the top eight predictors were retained. In the final stage, the core features identified in the first stage were compared with the top eight SHAP-ranked predictors from the second stage. The two approaches identified an identical set of predictors, which was subsequently entered into multivariable logistic regression to construct the final prediction model.

### 2.5 Statistical analysis

All analyses were performed using SPSS software (version 26.0) and R software (version 4.4.2). Patterns of missing data were evaluated using the md.pattern() function in the mice package, and missing values were assumed to be missing at random (MAR). Multiple imputation was subsequently performed using multivariate imputation by chained equations (MICE) with 10 imputed datasets (Mukherjee et al., 2023). Predictive mean matching, logistic regression, and polytomous regression were applied for continuous, binary, and categorical variables, respectively.

Continuous variables were assessed for normality using the Shapiro–Wilk test and are presented as mean ± standard deviation (SD) or median with interquartile range (IQR), as appropriate. Categorical variables are presented as frequencies and percentages. Between-group comparisons were performed using Student’s t test, the Mann–Whitney U test, the χ² test, or Fisher’s exact test, as appropriate. Correlations among predictors were evaluated using Spearman correlation coefficients and visualized using heatmaps.

Patients were randomly divided into training and validation cohorts at a ratio of 7:3. Feature selection was performed in the training set, and the final five variables were entered into the model. Multivariable logistic regression was conducted using the R package rms, with model fitting completed in the training set. During the model development phase, we assessed potential collinearity issues among the variables. All tolerance values exceeded 0.1, and the variance inflation factors (VIF) were below 10, indicating no significant collinearity. LASSO regression was implemented using the glmnet package with 10-fold cross-validation. Boruta analysis and RFE were performed according to their respective predefined procedures.

Model performance was evaluated in both the training and validation cohorts. Discriminative ability was assessed using ROC curve analysis with the pROC package, and AUC was used to quantify predictive performance (Lätti et al., 2016). Calibration performance was evaluated using calibration plots, the Brier score, and the Hosmer–Lemeshow goodness-of-fit test. Clinical utility was assessed using decision curve analysis (DCA), and model performance was further compared with that of qSOFA.

## 3 Results

### 3.1 Baseline characteristics

This study included 540 patients with PLA. The median age was 60 years (IQR, 51–68 years), including 316 men (58.52%) and 224 women (41.48%). Among these patients, 67 (12.4%) developed MODS, whereas the remaining 473 (87.6%) did not. All participants completed the relevant clinical and laboratory examinations. The baseline characteristics of the two groups are summarized in **Table 1**.

**Table 1:** Baseline characteristics of studied patients.

Items	Total patient cohort (n = 540)	Patients with PLA with MODS (n = 67)	Patients with PLA without MODS (n = 473)	P value	Validation set (n = 162)	Training set (n = 378)	P value
Age (years)	60.00 [51.00;68.00]	58.00 [46.50;66.50]	60.00 [52.00;68.00]	0.131	60.00 [52.00;66.00]	60.00 [51.00;68.00]	0.655
Sex, n (%)				1.000			0.894
Female	224 (41.48%)	28 (41.79%)	196 (41.44%)		66 (40.74%)	158 (41.80%)	
Male	316 (58.52%)	39 (58.21%)	277 (58.56%)		96 (59.26%)	220 (58.20%)	
Temperature (°C)	39.00 [38.10;39.60]	39.00 [37.80;39.20]	39.00 [38.20;39.80]	0.034	39.00 [37.92;39.60]	39.00 [38.30;39.60]	0.339
IKPLAS, n (%)				<0.001			0.453
No	511 (94.63%)	53 (79.10%)	458 (96.83%)		151 (93.21%)	360 (95.24%)	
Yes	29 (5.37%)	14 (20.90%)	15 (3.17%)		11 (6.79%)	18 (4.76%)	
Septicopyemia, n (%)				0.023			0.674
No	431 (79.81%)	46 (68.66%)	385 (81.40%)		127 (78.40%)	304 (80.42%)	
Yes	109 (20.19%)	21 (31.34%)	88 (18.60%)		35 (21.60%)	74 (19.58%)	
Viral hepatitis, n (%)				0.407			0.936
No	509 (94.26%)	65 (97.01%)	444 (93.87%)		152 (93.83%)	357 (94.44%)	
Yes	31 (5.74%)	2 (2.99%)	29 (6.13%)		10 (6.17%)	21 (5.56%)	
Liver cirrhosis, n (%)				0.378			1.000
No	528 (97.78%)	67 (100.00%)	461 (97.46%)		159 (98.15%)	369 (97.62%)	
Yes	12 (2.22%)	0 (0.00%)	12 (2.54%)		3 (1.85%)	9 (2.38%)	
Biliary disease, n (%)				0.308			0.907
No	430 (79.63%)	57 (85.07%)	373 (78.86%)		128 (79.01%)	302 (79.89%)	
Yes	110 (20.37%)	10 (14.93%)	100 (21.14%)		34 (20.99%)	76 (20.11%)	
Fatty liver, n (%)				1.000			1.000
No	523 (96.85%)	65 (97.01%)	458 (96.83%)		157 (96.91%)	366 (96.83%)	
Yes	17 (3.15%)	2 (2.99%)	15 (3.17%)		5 (3.09%)	12 (3.17%)	
Hypertension, n (%)				0.254			0.541
No	375 (69.44%)	42 (62.69%)	333 (70.40%)		116 (71.60%)	259 (68.52%)	
Yes	165 (30.56%)	25 (37.31%)	140 (29.60%)		46 (28.40%)	119 (31.48%)	
T2DM, n (%)				0.014			0.152
No	320 (59.26%)	30 (44.78%)	290 (61.31%)		88 (54.32%)	232 (61.38%)	
Yes	220 (40.74%)	37 (55.22%)	183 (38.69%)		74 (45.68%)	146 (38.62%)	
Hyperuricemia, n (%)				0.001			0.720
No	508 (94.07%)	56 (83.58%)	452 (95.56%)		151 (93.21%)	357 (94.44%)	
Yes	32 (5.93%)	11 (16.42%)	21 (4.44%)		11 (6.79%)	21 (5.56%)	
Abdominal operation, n (%)				0.469			0.070
No	464 (85.93%)	60 (89.55%)	404 (85.41%)		132 (81.48%)	332 (87.83%)	
Yes	76 (14.07%)	7 (10.45%)	69 (14.59%)		30 (18.52%)	46 (12.17%)	
WBC ( × 10 ^9^)	9.34 [6.72;12.93]	11.35 [8.09;16.46]	9.19 [6.58;12.51]	0.003	8.96 [6.47;12.46]	9.60 [6.89;13.42]	0.161
PLT ( × 10 ^9^)	227.00 [120.75;326.00]	125.00 [67.50;261.00]	237.00 [136.00;338.00]	<0.001	204.50 [107.25;294.00]	241.50 [125.00;338.00]	0.021
Hb (g/L)	115.00 [100.75;129.00]	114.00 [91.00;124.50]	115.00 [101.00;129.00]	0.264	114.50 [100.00;127.75]	115.00 [101.00;129.00]	0.612
CRP (mg/L)	109.59 [62.74;169.62]	135.00 [78.50;244.93]	106.51 [61.60;164.12]	0.006	107.18 [49.64;165.74]	114.58 [64.36;172.56]	0.440
ALP (U/L)	156.19 [114.00;217.08]	171.00 [111.14;222.38]	155.00 [115.00;216.77]	0.634	159.00 [117.75;211.32]	156.00 [112.50;224.50]	0.927
INR	1.11 [1.04;1.19]	1.16 [1.05;1.26]	1.11 [1.04;1.19]	0.032	1.11 [1.03;1.19]	1.11 [1.04;1.19]	0.995
Fibrinogen (g/L)	5.65 [4.65;6.63]	5.42 [4.01;6.04]	5.67 [4.68;6.64]	0.048	5.63 [4.48;6.72]	5.65 [4.74;6.59]	0.444
Abscess location, n (%)				0.681			0.164
Left lobe	154 (28.52%)	22 (32.84%)	132 (27.91%)		41 (25.31%)	113 (29.89%)	
Right lobe	297 (55.00%)	33 (49.25%)	264 (55.81%)		100 (61.73%)	197 (52.12%)	
Bilateral lobes	86 (15.93%)	12 (17.91%)	74 (15.64%)		20 (12.35%)	66 (17.46%)	
Caudate lobe	3 (0.56%)	0 (0.00%)	3 (0.63%)		1 (0.62%)	2 (0.53%)	
Number of abscesses, n (%)				0.331			0.844
1	308 (57.04%)	43 (64.18%)	265 (56.03%)		91 (56.17%)	217 (57.41%)	
2	231 (42.78%)	24 (35.82%)	207 (43.76%)		71 (43.83%)	160 (42.33%)	
3	1 (0.19%)	0 (0.00%)	1 (0.21%)		0 (0.00%)	1 (0.26%)	
Abscess diameter (mm)	60.00 [43.00;80.25]	64.00 [38.50;79.50]	59.50 [43.00;80.00]	0.782	59.50 [44.05;77.90]	60.00 [42.00;81.00]	0.983
Gas containing abscess, n (%)				0.119			0.576
No	478 (88.52%)	55 (82.09%)	423 (89.43%)		141 (87.04%)	337 (89.15%)	
Yes	62 (11.48%)	12 (17.91%)	50 (10.57%)		21 (12.96%)	41 (10.85%)	
Pathogen, n (%)				0.048			1.000
*Klebsiella pneumoniae*	523 (96.85%)	62 (92.54%)	461 (97.46%)		157 (96.91%)	366 (96.83%)	
*Escherichia coli*	17 (3.15%)	5 (7.46%)	12 (2.54%)		5 (3.09%)	12 (3.17%)	
Ascites, n (%)				<0.001			0.485
No	502 (92.96%)	46 (68.66%)	456 (96.41%)		153 (94.44%)	349 (92.33%)	
Yes	38 (7.04%)	21 (31.34%)	17 (3.59%)		9 (5.56%)	29 (7.67%)	
Perihilar abscess, n (%)				<0.001			0.544
No	527 (97.59%)	59 (88.06%)	468 (98.94%)		157 (96.91%)	370 (97.88%)	
Yes	13 (2.41%)	8 (11.94%)	5 (1.06%)		5 (3.09%)	8 (2.12%)	
Pulmonary infection, n (%)				0.005			1.000
No	466 (86.30%)	50 (74.63%)	416 (87.95%)		140 (86.42%)	326 (86.24%)	
Yes	74 (13.70%)	17 (25.37%)	57 (12.05%)		22 (13.58%)	52 (13.76%)	
Septic shock, n (%)				<0.001			0.281
No	468 (86.67%)	34 (50.75%)	434 (91.75%)		136 (83.95%)	332 (87.83%)	
Yes	72 (13.33%)	33 (49.25%)	39 (8.25%)		26 (16.05%)	46 (12.17%)	

IKPLAS, invasive *Klebsiella pneumoniae* liver abscess syndrome; T2DM, type 2 diabetes mellitus; WBC, white blood cell; PLT, platelet; Hb, hemoglobin; CRP, C-reactive protein; ALP, alkaline phosphatase; INR, International normalized ratio.

### 3.2 Correlation heatmap of predictor variables in the training cohort

The correlation matrix for variables in the training cohort is presented in **Supplementary Figure 2** and was used to evaluate the relationships among candidate predictors. Overall, most variables demonstrated weak correlations, as reflected by the predominance of light-colored regions in the heatmap. In contrast, deep blue and red regions indicated strong negative and positive correlations, respectively. Specifically, septicopyemia showed a significant positive correlation with WBC count, whereas septic shock was negatively correlated with PLT levels at admission. Spearman’s rank correlation analysis was used to calculate the correlation coefficients (ρ). The correlation analysis of the variables included in the final model is presented in **Supplementary Table 2**. The strongest pairwise correlation was observed between septic shock and PLT (ρ = −0.344), and the absolute values of all pairwise correlation coefficients were below 0.35, suggesting that there was no substantial multicollinearity among the variables included in the final model.

### 3.3 Screening of predictive factors

**Figure 3:**
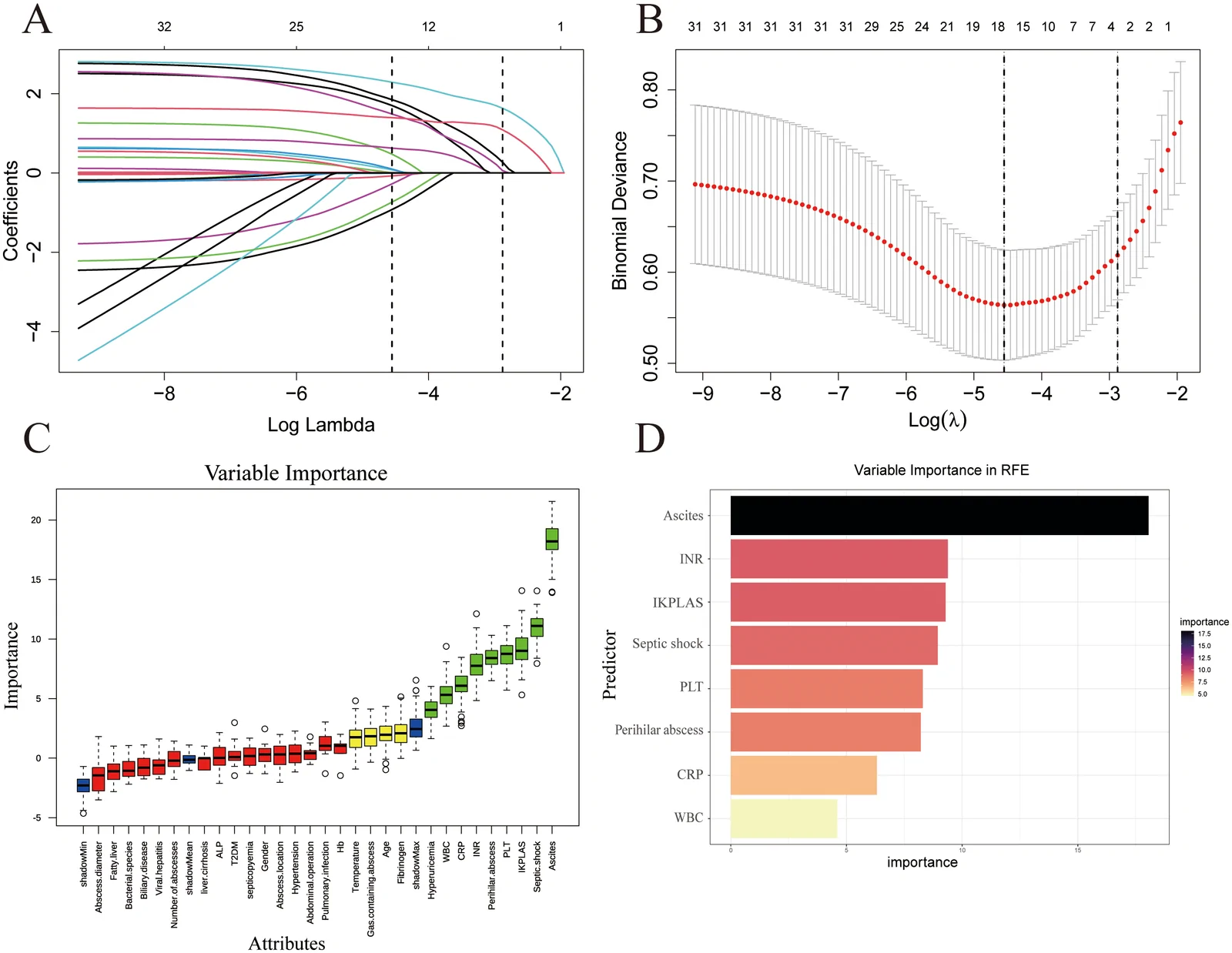
Predictor screening results. **(A)** LASSO regression model screening variable trajectories; **(B)** Factor screening based on the LASSO regression model, with the left dashed line indicating the best lambda value for the evaluation metrics (lambda. min) and the right dashed line indicating the lambda value for the model where the evaluation metrics are in the range of the best value by one standard error (lambda.1se); **(C)** Boruta; **(D)** Variable importance ranking based on RFE.

**Figure 4:**
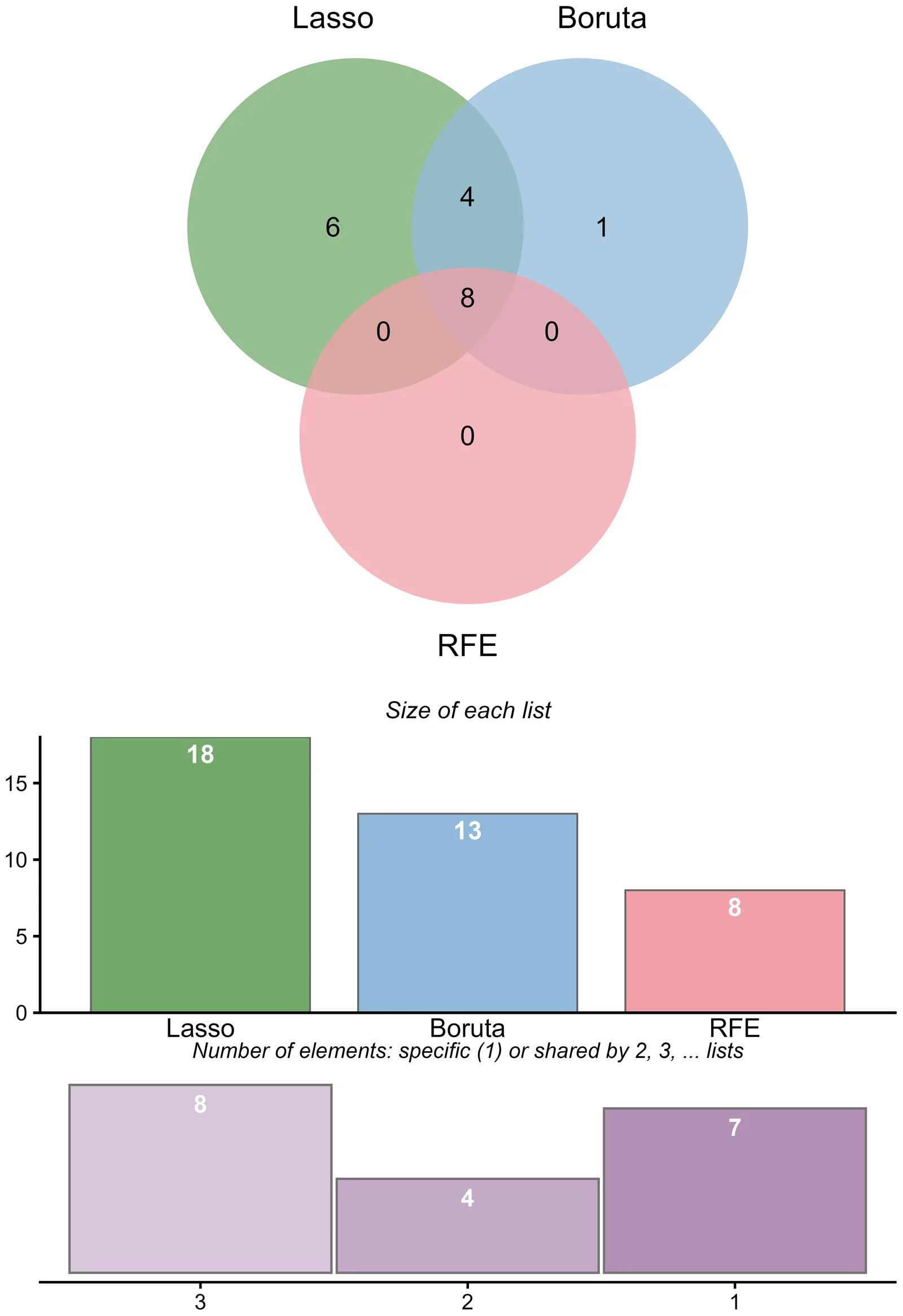
Venn diagram of the features selected by LASSO, Boruta, and RFE.

A total of 540 patients with PLA were enrolled and randomly divided in a 7:3 ratio into a training set (n=378) and a validation set (n=162). No significant differences in baseline characteristics were observed between the two cohorts. To identify candidate predictors associated with MODS, we initially performed least absolute shrinkage and selection operator (LASSO) regression analysis. Using 10-fold cross-validation, the optimal penalty parameter was determined at λ_min, corresponding to the minimum binomial deviance. This approach identified 18 potential predictors, including temperature, age, viral hepatitis, fatty liver, T2DM, prior abdominal surgery, gas-containing abscess, pathogen, ascites, septic shock, IKPLAS, PLT, perihilar abscess, INR, CRP, WBC, hyperuricemia, and septicopyemia (**Figures 3A, B**). To enhance robustness, we next used the Boruta algorithm to confirm important variables by comparing their importance with that of shadow features. Boruta retained 13 key predictors, including ascites, septic shock, IKPLAS, PLT, perihilar abscess, INR, CRP, WBC, hyperuricemia, fibrinogen, age, gas-containing abscess, and temperature (**Figure 3C**). All candidate variables were then further assessed using RFE, which iteratively fits models and removes the least informative variables to derive an optimal feature subset. RFE ultimately selected 8 predictors with the strongest predictive value (**Figure 3D**). The overlapping variables identified consistently across LASSO, Boruta, and RFE were ascites, septic shock, IKPLAS, PLT, perihilar abscess, INR, CRP, and WBC. These predictors were defined as the key predictor set and used to develop the diagnostic model for MODS in patients with PLA (**Figure 4**).

**Figure 5:**
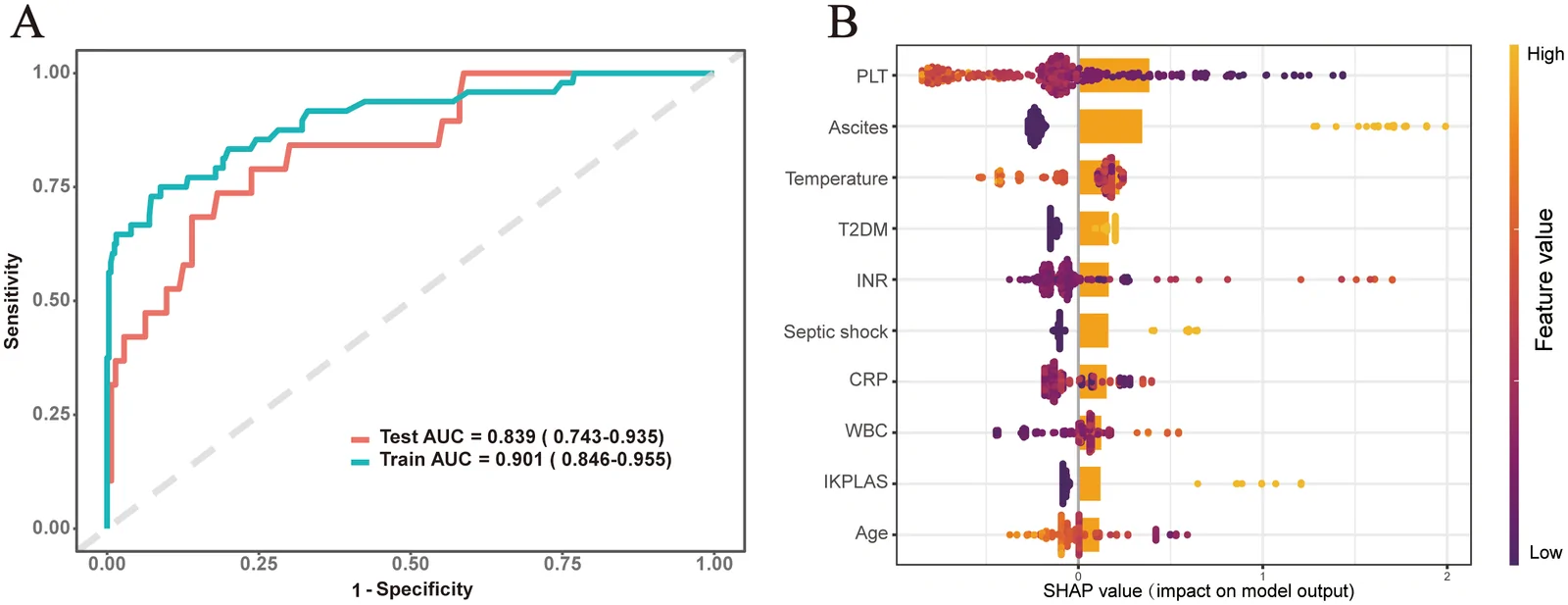
**(A)** ROC curves and AUCs of the model in the training and test sets; **(B)** SHAP summary plot showing feature importance ranked by mean absolute SHAP values, along with the direction and magnitude of each feature’s effect on the predicted risk.

To further validate the importance of these variables, an XGBoost model was constructed using the combined variables identified by the three feature-selection methods. SHAP values were then used to quantify and rank feature contributions (**Figure 5**). Notably, the eight highest-ranking SHAP features were identical to the consensus predictor set identified above. Accordingly, this feature set was subsequently entered into the multivariable logistic regression model for final model construction. In multivariable analysis, ascites, septic shock, IKPLAS, PLT, and INR were independently associated with MODS (**Table 2**).

**Table 2:** Logistic regression results for risk factors associated with MODS in patients with PLA.

Factors	OR (95%CI)	P value
IKPLAS	7.667 (2.397–24.526)	<0.001
Ascites	13.834 (5.280–36.245)	<0.001
INR	6.983 (1.274–38.287)	<0.05
PLT	0.997 (0.994–1.000)	<0.05
Septic shock	4.662 (1.935–11.235)	<0.001

IKPLAS, invasive *Klebsiella pneumoniae* liver abscess syndrome; INR, International normalized ratio; PLT, platelet.

### 3.4 Development and validation of the nomogram

**Figure 6:**
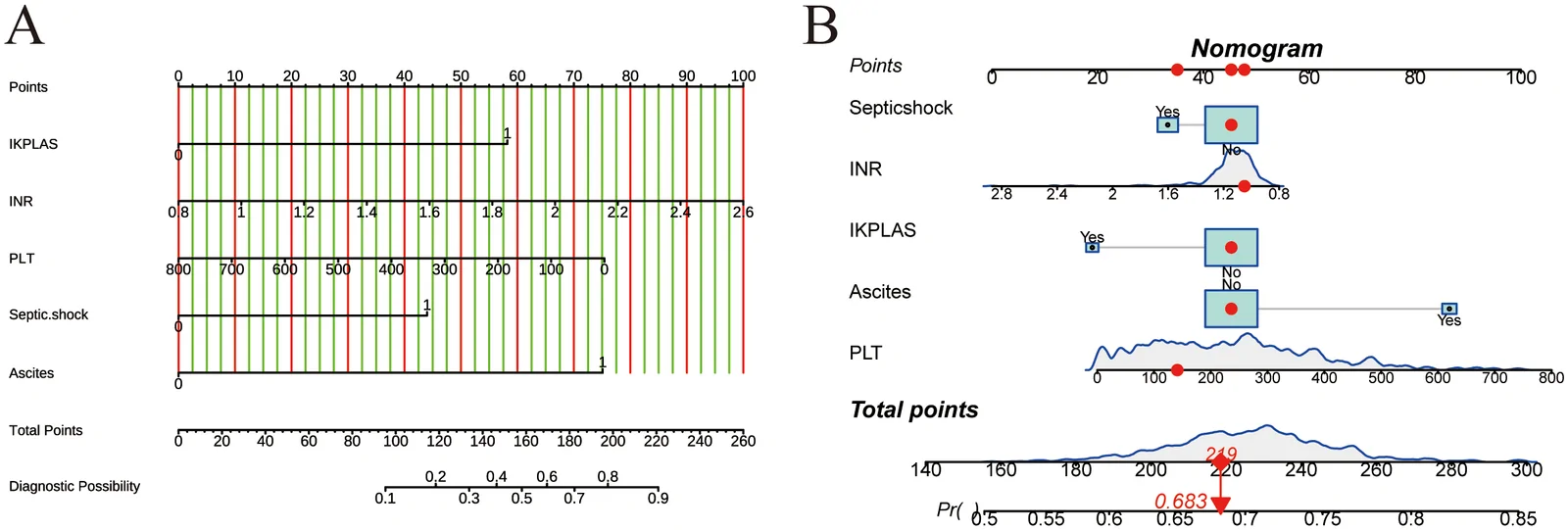
**(A)** IKPLAS, INR, PLT, septic shock, and ascites were included as risk factors in the nomogram prediction model; **(B)** Dynamic nomogram.

An individualized risk prediction model was established based on five independent risk factors to estimate the probability of MODS in patients with PLA (**Figure 6**). To facilitate clinical application and improve interpretability, these predictors were integrated into a nomogram.

### 3.5 Predictive accuracy

**Figure 7:**
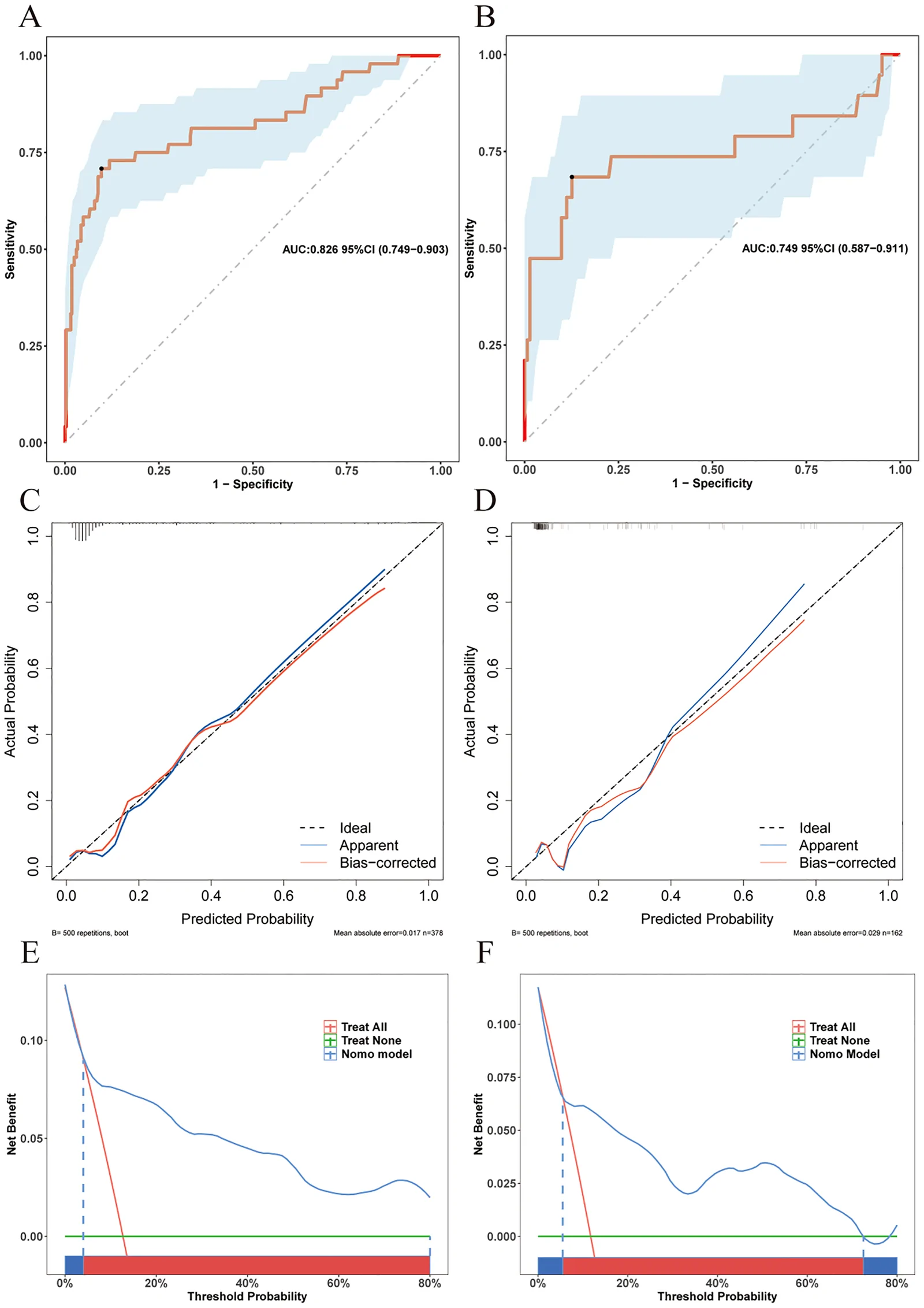
ROC curve **(A)**, calibration curves **(C)**, and decision curve analysis **(E)** for cognitive impairment prediction in the training cohort. ROC curve **(B)**, calibration curves **(D)**, and decision curve analysis **(F)** for cognitive impairment prediction in the validation cohort.

The nomogram demonstrated good discriminative performance in both the training and validation cohorts, with an AUC of 0.826 in the training set (95% CI: 0.749–0.903) and 0.749 in the validation set (95% CI: 0.587–0.911) (**Figures 7A, B**). Calibration curves showed close agreement between the predicted and observed probabilities of MODS, with the curves closely approximating the ideal diagonal reference line (**Figures 7C, D**). DCA further demonstrated the potential clinical utility of the nomogram. As shown in **Figures 7E, F**, the model provided a higher net benefit across a wide range of threshold probabilities, approximately 5%–80% in the training cohort and 5%–75% in the validation cohort. Within these threshold ranges, the nomogram consistently outperformed both the “treat-all” and “treat-none” strategies, supporting its value in clinical decision-making.

### 3.6 Clinical applicability

To further assess the clinical applicability of the nomogram, clinical impact curve (CIC) analysis and net reduction curve analysis were performed. The CICs demonstrated broadly consistent trends between the training and validation cohorts (**Supplementary Figure 3**), indicating favorable model stability and potential generalizability in clinical settings. Net reduction curve analysis showed that, compared with the “treat-all” strategy, the model provided relatively limited reductions in unnecessary interventions at lower decision thresholds. However, at higher threshold probabilities, the nomogram substantially reduced avoidable intensified management (**Supplementary Figure 4**). Overall, the CIC and net reduction analyses support that the model offers not only strong predictive performance but also meaningful clinical decision support.

### 3.7 Model comparison and incremental predictive value

To compare the nomogram developed in this study (ModB) with the traditional qSOFA score (ModA) for predicting MODS in patients with pyogenic liver abscess, we plotted ROC curves and calculated AUCs in both the training and validation sets to assess discrimination, and used the DeLong test to compare AUCs between models. Moreover, DCA was performed to evaluate clinical net benefit across different threshold probabilities. In addition, to quantify improvement in risk reclassification and incremental discrimination of the nomogram over qSOFA, we calculated the categorical NRI and IDI, using risk categories of predicted probability <0.10, 0.10–0.30, and ≥0.30.

**Figure 8:**
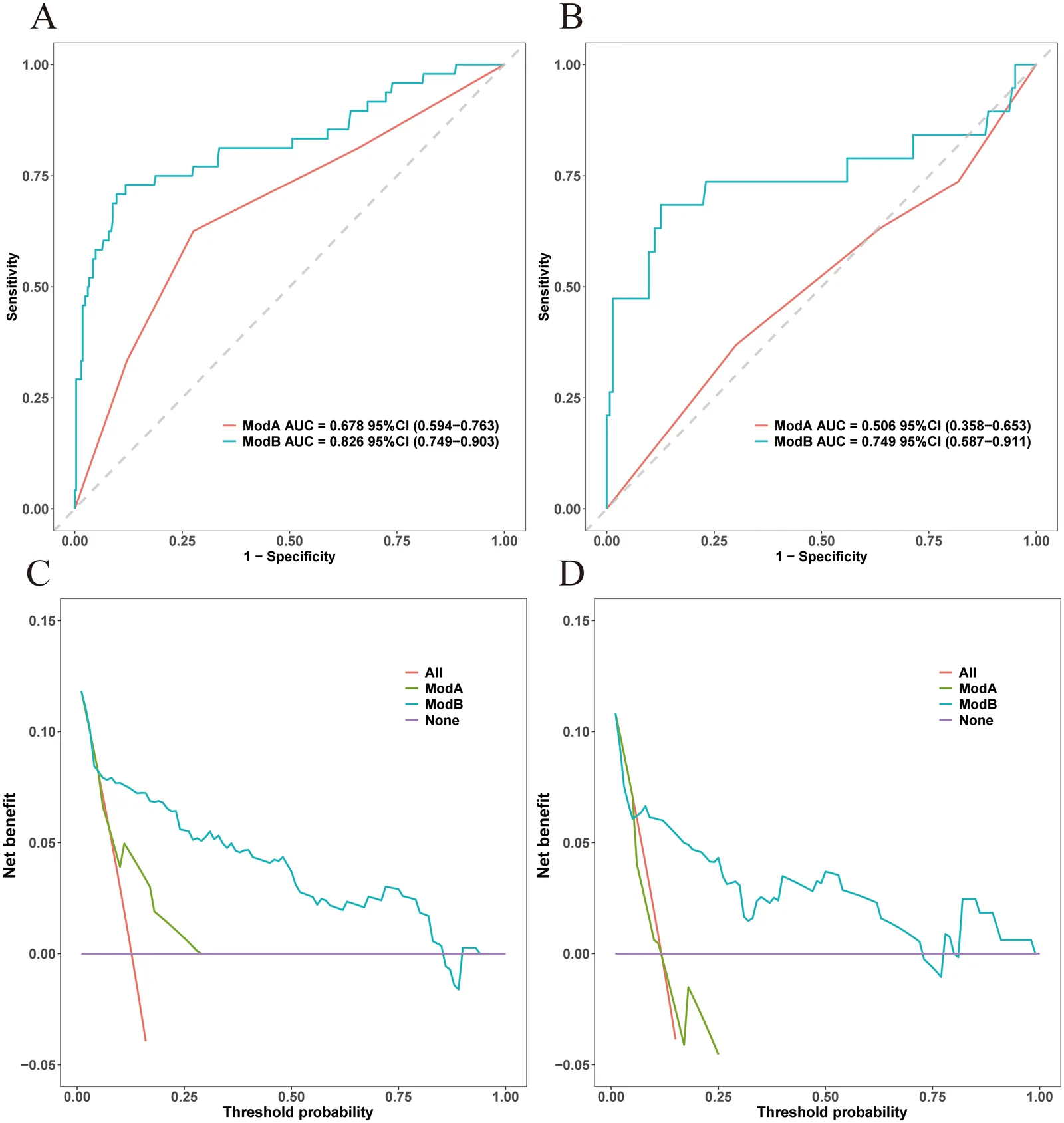
**(A, B)** AUC comparison between the nomogram model (ModB) and the traditional qSOFA score (ModA) in the training and validation sets; **(C, D)** DCA comparing the nomogram model (ModB) and the traditional qSOFA score (ModA) in the training and validation sets.

ROC analyses showed that the nomogram outperformed qSOFA in both datasets. In the training set, the nomogram achieved an AUC of 0.826 (95% CI: 0.749–0.903), compared with 0.678 (95% CI: 0.594–0.763) for qSOFA, with a significant difference according to the DeLong test (*P =* 0.011). Similarly, in the validation set, the nomogram consistently demonstrated better discrimination than qSOFA (**Figures 8A, B**; **Table 3**). DCA showed that the nomogram provided greater net benefit than qSOFA within the clinically relevant threshold probability range of 0.10–0.30 (**Figures 8C, D**). Reclassification analyses additionally confirmed the incremental predictive value of the nomogram. In the training cohort, the NRI and IDI values were 0.8803 (95% CI: 0.6323–1.1283) and 0.2851 (95% CI: 0.1921–0.3781), respectively. In the validation cohort, the NRI and IDI values were 1.0604 (95% CI: 0.6429–1.4778) and 0.3142 (95% CI: 0.1706–0.4579), respectively. These findings indicate that the nomogram substantially improved risk reclassification and incremental discrimination compared with qSOFA.

**Table 3:** Comparison of deLong's test between nomogram model and traditional scoring models in training set and validation set.

	Model A AUC (95%CI)	Model B AUC (95%CI)	Test method	Z-value	P-value	ΔAUC (95% CI)
Training set	qSOFA 0.678 (0.594-0.763)	Nomogram 0.826 (0.749-0.903)	DeLong's test	-2.546	0.011	0.148(0.034–0.261)
Validation set	qSOFA 0.506 (0.358-0.653)	Nomogram 0.749 (0.587-0.911)	DeLong's test	-2.371	0.018	0.243(0.042–0.444)

AUC, area under curve; qSOFA, quick Sequential Organ Failure Assessment.

## 4 Discussion

In the present study, multivariable logistic regression analysis identified IKPLAS, septic shock, ascites, INR, and PLT as independent predictors of MODS in patients with PLA. Based on these variables, we developed and validated a nomogram for admission-based and early in-hospital risk stratification that enables individualized estimation of MODS probability. The model demonstrated stable discrimination, good calibration, and favorable clinical utility, while also outperforming the qSOFA score. To our knowledge, this is the first study to establish and validate a dedicated prediction model for MODS in patients with PLA.

Collectively, these five predictors reflect several key pathophysiological mechanisms linking severe infection to organ dysfunction, including pathogen invasiveness, circulatory instability, impaired coagulation, and systemic inflammatory dysregulation. This finding is consistent with previous evidence showing that infection may trigger a dysregulated host response that ultimately culminates in organ dysfunction (Yang et al., 2020; Tang et al., 2024). Among these predictors, septic shock may represent the most direct clinical indicator of impending MODS. According to the Sepsis-3 consensus, sepsis-related organ dysfunction results primarily from a dysregulated host response rather than the infection itself, whereas septic shock represents a more severe subtype characterized by profound circulatory, cellular, and metabolic abnormalities that markedly increase the risk of death and organ failure (Singer et al., 2016; Cecconi et al., 2018). Its pathophysiological basis involves impaired microcirculatory perfusion, endothelial injury, and disruption of barrier function, thereby accelerating the progression to multiple organ dysfunction (Raia and Zafrani, 2022; Tang et al., 2024). Therefore, in clinical practice, once a patient with PLA develops signs of shock, clinicians should maintain a high index of suspicion for impending organ failure and promptly initiate ICU-level monitoring and organ-supportive therapies to reduce the risk of progression to MODS (Evans et al., 2021).

IKPLAS also emerged as an independent risk factor for MODS, suggesting that pathogen phenotype plays a critical role in determining disease severity and adverse clinical outcomes. In Asian populations, hypervirulent *Klebsiella pneumoniae* (hvKP) is recognized as the predominant pathogen responsible for IKPLAS and is well known for its propensity to cause dissemination and metastatic infections, including endophthalmitis, central nervous system infection, and septic pulmonary embolism (Wang et al., 2023). These manifestations indicate that infection is frequently no longer confined to the liver and is more likely to trigger systemic inflammatory responses and subsequent organ injury (Siu et al., 2012; Gu et al., 2025; Wang et al., 2025). In recent years, the global incidence of KPLA has increased substantially, with 15%–25% of patients developing extrahepatic metastatic infections (Butt et al., 2021). Our previous work focused on the early identification of invasive syndrome in patients with liver abscess and established a prediction model for IKPLAS-related risk stratification (Zhang et al., 2025), which provided an important foundation for the present study. Taken together, these findings suggest that management of patients with suspected or confirmed IKPLAS should extend beyond conventional abscess treatment and include active surveillance for systemic complications. This includes prompt assessment of visual function and neurological status, with targeted imaging studies performed when clinically indicated. Antimicrobial therapy should be dynamically optimized based on microbiological evidence and the risk of antimicrobial resistance, to reduce the likelihood of metastatic infection and progression to severe disease (Gu et al., 2025).

Elevated INR and reduced PLT were also identified as independent predictors, highlighting the important role of the coagulation–inflammation axis in the progression of MODS. Sepsis is known to induce a spectrum of coagulation abnormalities ranging from sepsis-induced coagulopathy (SIC) to disseminated intravascular coagulation (DIC). Conventional coagulation markers such as PLT and INR are commonly used for early recognition of this process because they reflect endothelial injury, coagulation factor consumption, microthrombus formation, and impaired microcirculatory perfusion, all of which are closely associated with organ failure (Giustozzi et al., 2021; Iba et al., 2021; Tsantes et al., 2023). Abnormal PLT levels may reflect both inflammatory activation and coagulation dysfunction (Steinbach et al., 2024; Adelman et al., 2025; Li et al., 2025). Thrombocytopenia is particularly common in critically ill patients, with the reported incidence of newly developed thrombocytopenia during ICU stay ranging from 13.0% to 44.1% (Hui et al., 2011), and has been associated with multiple organ failure and mortality (Pène et al., 2025). In addition to reflecting disease severity and coagulation consumption, reduced PLT may contribute to organ injury through dysregulated immune responses and altered platelet–endothelial interactions (Cheng et al., 2023; Alqeeq et al., 2025). Previous studies have also shown that PLT and other coagulation-related parameters have good predictive value for disease severity and prognosis in elderly patients with sepsis (Adelman et al., 2025).

INR reflects abnormalities in the extrinsic coagulation pathway and may indicate impaired synthesis or increased consumption of coagulation factors (Iba et al., 2021; Tsantes et al., 2023). Previous ICU-based studies have demonstrated that elevated INR is independently associated with 28-day mortality and may serve as a useful marker for early risk stratification at admission (Liu et al., 2021). Taken together, elevated INR and reduced PLT may reflect progressive coagulopathy, impaired hepatic synthetic function, and microcirculatory dysfunction under severe infectious stress, thereby contributing to the development of MODS. Therefore, these abnormalities should prompt intensified monitoring and timely assessment for the development or progression of SIC and DIC.

Ascites was another independent predictor identified in this study and may reflect impaired hepatic reserve and disrupted fluid homeostasis, both of which reduce the ability to tolerate severe infectious stress and increase susceptibility to critical illness (European Association for the Study of the Liver (EASL), 2018). Current guidelines indicate that severe microbial infections such as sepsis can precipitate acute-on-chronic liver failure (ACLF), a syndrome characterized by acute decompensation on the background of chronic liver disease, accompanied by failure of one or more organ systems and high short-term mortality (European Association for the Study of the Liver (EASL), 2023). Accordingly, the presence of ascites not only reflects impaired hepatic reserve but may also signify a state of increased susceptibility to organ dysfunction under infectious stress. Furthermore, the presence of pleural effusion and ascites often suggests infection spread or the development of liver abscess–related complications, and both have been reported to be significantly associated with adverse outcomes (Lee et al., 2008; Petri et al., 2019). Previous studies have demonstrated that pleural effusion is frequently associated with sepsis and MODS, and its development may be related to increased capillary permeability and fluid redistribution driven by the systemic inflammatory response, thereby precipitating or aggravating respiratory dysfunction and even progressing to respiratory failure (Petri et al., 2019). By comparison, ascites in patients with PLA may additionally suggest abscess enlargement, impending abscess rupture, or biliary tract involvement (Huang et al., 2022), all of which substantially increase the risk of clinical deterioration. This may be particularly important in immunocompromised individuals, in whom pleural effusion and ascites may serve as early warning signs of sepsis and impending multiple organ failure (Hao et al., 2025). It should be noted that pleural effusion was not included in the variable set of the present study, which may have led to an insufficient assessment of its association with prognosis and therefore represents one of the limitations of this study. Collectively, the presence of pleural effusion or ascites in patients with PLA should be regarded as a high-risk signal. In such cases, dynamic monitoring of hepatic and renal function, nutritional status, and the effectiveness of infection control should be intensified, and early, targeted interventions should be implemented to reduce the risk of progression to MODS.

Previous studies have reported that cardiovascular comorbidities, pleural effusion, and pulmonary infection are associated with an increased risk of MODS in patients with PLA (Yin et al., 2021). Building on these findings, the present study incorporated IKPLAS, septic shock, ascites, INR, and PLT into a diagnostic model to facilitate early identification and risk assessment of MODS. Although pulmonary infection was associated with MODS in the univariate analysis, it did not remain an independent predictor after multivariable adjustment. This may be because pulmonary infection primarily reflects greater infectious burden or systemic involvement, whereas septic shock, elevated INR, and reduced PLT more directly represent circulatory failure, coagulation–inflammation dysregulation, and subsequent organ injury. Nevertheless, pulmonary infection remains clinically important, as the lungs are often affected early during sepsis-related organ dysfunction. Pulmonary endothelial barrier disruption, increased capillary permeability, alveolar congestion, and impaired surfactant function may promote the development of acute respiratory distress syndrome (ARDS) and amplify systemic inflammation (Bos and Ware, 2022; Sun et al., 2023; Su et al., 2024).

In the present study, the nomogram achieved higher AUC values than the conventional qSOFA score in both the training and validation cohorts and provided greater net benefit across a range of threshold probabilities. The NRI and IDI analyses further demonstrated improvements in risk reclassification and incremental discrimination. These advantages may be attributable to the inclusion of pathogen invasiveness and coagulation dysfunction, which are not captured by qSOFA, thereby enabling a more comprehensive assessment of the pathophysiological continuum from infection severity and host response to organ injury. These findings suggest that, in patients with PLA, reliance solely on simplified physiological scores may underestimate the risk in some high-risk individuals, whereas integrating pathogen-related and coagulation-related indicators may allow earlier and more accurate risk stratification (Singer et al., 2016; Tsantes et al., 2023). However, this study was unable to further compare the nomogram with more comprehensive clinical scoring systems, such as APACHE II and NEWS2, mainly because several key variables were incompletely documented in the retrospective electronic medical records. Restricting the analysis to patients with complete data could have substantially reduced the sample size and increased the risk of selection bias.

Despite these findings, several limitations of the present study should be acknowledged. First, because of the retrospective study design, the effects of missing data, information bias, and selection bias could not be completely avoided. In addition, several potential confounding factors were not included in the analysis, such as the specific pathogen spectrum, the selection of empirical antimicrobial therapy, and differences in early interventions, including the timing of drainage, which may have affected both clinical outcomes and model performance. Second, the relatively small sample size and single-center design may limit the generalizability of the findings. Several variables were also susceptible to incomplete documentation and potential measurement error, particularly certain clinical and imaging indicators.

Furthermore, because SOFA subscores for some organ systems were incompletely documented, the identification of organ involvement in MODS partially relied on medical record descriptions and the use of organ-supportive therapies, which may have introduced misclassification bias. Furthermore, because the determination of IKPLAS depends on microbiological and imaging evidence, it may not always be available immediately at the time of admission. Therefore, the complete model may be more suitable for dynamic risk stratification based on admission data and early hospitalization information than for immediate risk prediction in the emergency department. Septic shock is also an important clinical marker of severe infection and organ dysfunction, and its inclusion may, to some extent, reduce the novelty of the model. However, the purpose of the model was to integrate multiple clinical and laboratory variables to provide an individualized and quantitative assessment of the risk of MODS in patients with PLA, rather than to emphasize the predictive value of any single factor.

Moreover, the applicability of the model in patients with extreme variable values or in specific subgroups, such as older patients and those with severe underlying diseases, remains to be further evaluated. Therefore, future multicenter, prospective studies with larger sample sizes and broader variable dimensions are warranted. External validation and dynamic updating across different pathogen distributions and antimicrobial resistance backgrounds will also be necessary to further assess the robustness and generalizability of the model. Ultimately, these efforts may provide stronger support for individualized risk management and more precise treatment strategies in patients with liver abscess.

## 5 Conclusions

In summary, we developed and validated a nomogram for predicting the risk of MODS in patients with PLA. The model incorporated IKPLAS, septic shock, ascites, INR, and PLT as key independent predictors and demonstrated good discrimination, calibration, and clinical utility.

## Data Availability

The original contributions presented in the study are included in the article/**Supplementary Material**. Further inquiries can be directed to the corresponding author.

## Supplementary material

Supplementary Figure 1 Correlation matrix of variables in the training cohorts.

Supplementary Figure 2 Clinical impact curve (CIC) illustrates the clinical utility of the nomogram. **(A)** Represent the predictions in the training set; **(B)** Represent the predictions in the validation set.

Supplementary Figure 3 Net reduction curve illustrates the clinical utility of the nomogram. **(A)** Represent the predictions in the training set; **(B)** Represent the predictions in the validation set.
