# Supplementary material for: Clinical characteristics of pyogenic liver abscess patients with multiple organ dysfunction syndrome and development and validation of a predictive model
Source: Front Cell Infect Microbiol. 2026 Aug 20;16:1828061. doi: 10.3389/fcimb.2026.1828061 (PMC13522978; doi:10.3389/fcimb.2026.1828061)
Supplement: Supplementary file 1 [file Table1.docx]

**Table S1**

**Supplementary Table S1.** Early clinical interventions in the non-MODS and MODS groups

| Variable | Non-MODS group (n = 473) | MODS group (n = 67) | P value |
| --- | --- | --- | --- |
| Percutaneous drainage within 24 h, n (%) | 255 (53.9) | 33 (49.3) | 0.474 |
| Empirical broad-spectrum antibiotics, n (%) | 392 (82.9) | 61 (91.0) | 0.109 |

Note: P values were calculated using the chi-square test or Fisher's exact test, as appropriate.
