# Supplementary material for: Clinical characteristics of pyogenic liver abscess patients with multiple organ dysfunction syndrome and development and validation of a predictive model
Source: Front Cell Infect Microbiol. 2026 Aug 20;16:1828061. doi: 10.3389/fcimb.2026.1828061 (PMC13522978; doi:10.3389/fcimb.2026.1828061)
Supplement: Supplementary file 2 [file Table2.docx]

**Table S2**

**Supplementary Table S2.** Spearman correlation coefficients among final predictors

| Variable | Ascites | Septic shock | IKPLAS | PLT | INR |
| --- | --- | --- | --- | --- | --- |
| Ascites | 1.000 | 0.190 | 0.063 | -0.107 | 0.090 |
| Septic shock | 0.190 | 1.000 | 0.148 | -0.344 | 0.175 |
| IKPLAS | 0.063 | 0.148 | 1.000 | -0.083 | -0.081 |
| PLT | -0.107 | -0.344 | -0.083 | 1.000 | -0.152 |
| INR | 0.090 | 0.175 | -0.081 | -0.152 | 1.000 |

Note: Values are Spearman's rho (ρ) coefficients calculated using pairwise complete observations. All pairwise absolute correlation coefficients were below 0.35.
